# Lingual tonsil pseudolymphoma and obstructive sleep apnea

**DOI:** 10.1016/S1808-8694(15)30670-4

**Published:** 2015-10-19

**Authors:** Patrícia Eiko Yamakawa, Elvis Henrique Santos Andrade, Carlos Hirokatsu Watanabe-Silva, Leopoldo Luiz Dos Santos Neto

**Affiliations:** 1 Medical student, Universidade de Brasilia - UnB; 2 Medical student, Universidade de Brasilia - UnB; 3 Medical student, Universidade de Brasilia - UnB; 4 Doctorate in molecular pathology, Universidade de Brasília. Adjunct professor of clinical medicine, Universidade de Brasília- UnB

**Keywords:** hyperplasia, obstructive, sleep apnea, tongue, tonsil

## INTRODUCTION

Lingual tonsils are the most neglected element of Waldeyer's ring; they cannot be visualized in a plain oropharyngeal examination. Lingual tonsillar hypertrophy (LTH) is frequently asymptomatic and underdiagnosed, but may be associated with obstructive sleep apnea (OSA); it is also included in the differential diagnosis of tongue base lymphoma.^1^,^2^ We present a case report and a review of the literature.

## CASE REPORT

A male patient aged 36 years complained of snoring and insomnia. There was a history of nasal voice and serous otitis media since childhood, including placement of ventilation tubes (the last one in 2003). The patient underwent tonsillectomy at age 7 years and adenoidectomy at age 33 years. The patient smoked (15 years-pack) but did not consume alcoholic beverages. At the physical examination the patient was obese (BMI = 31.9 kg/m2), and there were no visible oropharyngeal signs.

**Figure 1:**
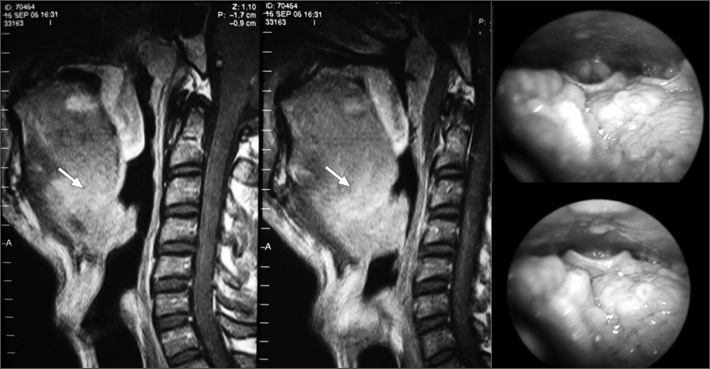
Lingual tonsil hyperplasia - Laryngoscopic and magnetic resonance images demonstrating lingual tonsil hyperplasia.

Polysomnography demonstrated fragmented sleep with microawakenings and snoring; sleep efficiency was 77%; the apnea-hypopnea index was 10 events per hour; minimum O2 saturation was 86%. A diagnosis of OSA was made based on clinical and polysomnographic findings. Magnetic resonance imaging (MRI) demonstrated a hypopharyngeal 89 mm^2^ nodule measuring 4 × 3.5 × 2.5 cm, located on the epiglottic valeculla and increasing the thickness of the epiglottis (Fig. 1). Videolaryngoscopy confirmed this finding (Fig. 1). Facial sinus, thoracic and abdominal computed tomography revealed no significant findings.

A biopsy yielded an initial diagnosis of B immunophenotype non-Hodgkin's lymphoma, based on which the patient underwent four chemotherapy sessions. A second review of the slide, however, concluded that the lesion was in fact a reactional follicular lymphoid hyperplasia, and chemotherapy was discontinued. A bone marrow biopsy was normal.

The patient refused surgery; he was encouraged to lose weight. Up to the present, there has been no improvement.

## DISCUSSION

The pathophysiology of LTH is not yet clear; atopy, smoking, and chronic lingual tonsil infection may contribute to its development.^1^ The most commonly admitted cause of LTH is compensating hyperplasia following tonsillectomy and/or adenoidectomy; two thirds of LTH patients report having had these procedures.^1^

A diagnosis of LTH requires an assessment of the tongue base and hypopharynx with indirect laryngoscopy or an optic fiber laryngoscope. Image exams, such as MRI, are useful for detecting and establishing the size of lingual tonsils.

The differential diagnosis of LTH includes an ectopic thyroid, thyroglossal duct cysts, demoid cysts, angiomas, lymphangiomas, adenomas, fibromas, papillomas, squamous cell carcinomas, minor salivary gland tumors of the base of the tongue, and lymphomas.^1^ There are also reports in the literature of non-Hodgkin's lymphoma, Burkitt's lymphoma, and MALT lymphoma involving lingual tonsils.^2^, ^3^, ^4^

LTH in adults is often asymptomatic, and may be detected only during procedures involving the airway, causing difficult tracheal intubation.^1^ However, lingual tonsils may be significantly increased, filling the valeculla and displacing the epiglottis posteriorly. LTH is also associated with dyspnea, odynophagia, dysphagia, chronic coughing, hoarseness, and recurring epiglottic abscesses.^1^,^5^

LTH is an uncommon cause of OSA.^1^,^5^ It may occur in children in association with nocturnal pharyngeal collapse, especially in Down's syndrome.^5^ LTH-caused OSA is rare in adults; there are four such reports in the literature.^1^,^5^,^6^ The authors suggest that significant LTH and obesity led to OSA in this patient.

Lingual tonsillectomy is recommended in cases with any degree of airway obstruction that impairs breathing, including the treatment of OSA. Results are good in these cases.^5^

## FINAL COMMENTS

LTH is an uncommon and little diagnosed finding. It may, however, cause significant symptoms and affect the quality of life of patients. LTH is part of the differential diagnosis of OSA and lymphomas of the base of the tongue, and should be taken into account when these cases are investigated.
